# Editorial: Women's cycling: specificities, situation and perspectives

**DOI:** 10.3389/fspor.2024.1498761

**Published:** 2024-10-02

**Authors:** Solene Le Douairon Lahaye, Fabien Ohl

**Affiliations:** ^1^ Laboratory Movement, Sport and Health Sciences (M2S-EA7470), UFR APS, University of Rennes, Rennes, France; ^2^ Faculty of Social and Political Sciences, Sport Sciences Institute (ISSUL), University of Lausanne, Lausanne, Switzerland

**Keywords:** women, cycling, situation, specificity, perspectives

**Editorial on the Research Topic**
Women’s cycling: specificities, situation and perspectives

Women's cycling: specificities, situation and perspectives is a *Frontiers in Sports and Active Living—Elite Sports and Performance Enhancement* Research Topic aimed to describe precisely the specificities and the current situation of women's cycling.

For several years, women's cycling has been in full swing. The development of the practice for all women on the one hand and the professionalization of elite women athletes on the other hand represents a major challenge for the Union Cycliste Internationale (UCI) as evidenced by the “UCI Women in cycling—Best practice guide”.

The situation of women's cycling is improving, but must continue taking into account its specificities and the discrepancy between the status of women's and men's cycling.

Accordingly, the objective of this Research Topic was to collect data pertaining to the distinctive characteristics of women's cycling (at both amateur and elite levels) from a comprehensive and multifaceted perspective.

This Research Topic accepted 4 articles for publication (2 original research, 1 brief research report and 1 perspective articles) written by a total 33 contributing authors.

Addressing the specifics of women's cycling cannot be done without discussing female physiology and the impact of menstrual cycles (or oral contraception) on performance. Indeed, the fluctuations in hormones during the menstrual cycles (natural or not) generate numerous variables that impact the response to exercise, the response to training sessions and thus to performance (1, 2). In this context, some authors posit that coaches and athletes could derive significant benefits from adapting and individualizing training programmes according to the menstrual cycle (2). In original research, Carlin et al. proposed a novel approach to determine the relation between the menstrual cycle and athletes’ training responses of elite women cyclists. In consideration of the model put forth by the authors, it is possible to state that elite women cyclists may derive benefit from engaging in light-to-moderate training during the menstrual phase and more intensive training immediately thereafter. This novel approach encourages further investigation to optimize individual training strategies taking into account the female physiology.

In this way, Sawai et al. present an original article that determine the impact of cycling exercise on cell-free DNA (a potential biomarker of injury level, inflammation, cellular stress and cell death) depending of menstrual cycle phase. This study provides new insights into the impact of the menstrual cycle on the effects of exercise and confirms the relevance of individualization of training during the menstrual cycle in cyclists and more widely among women athletes.

The development of women's cycling depends on expanding the competition calendar. It would be beneficial to see an increase in both one-day but also more multi-stages competitions. The most significant development was the reinstatement of the women's Tour de France in 2022. Due to the high cardiac workload involved, this type of ultra-endurance event has the potential to induce an exercise-induced cardiac fatigue which could have negative impact in performance and health (3). In a brief report, Menard et al. describe the evolution of the cardiac function assessed by echocardiography and the autonomic cardiac function assessed by the HRV during a multi-stage event in a well-trained female cyclist. Performed at a low intensity, no indications of fatigue were observed during the multi-stage cycling event. However, further research is required to evaluate the impact of this type of event in competitive conditions.

Despite the considerable advancements witnessed in the domain of professional women's cycling in recent times, the prevailing working conditions remain suboptimal. This can potentially exert a detrimental influence on the mental well-being of the athletes. In their perspective paper, Colangelo et al., provide an overview of psychiatric concerns prevalent among women engaged in professional cycling. Based on this assessment, they put forth a series of recommendations aimed at fostering gender equality and, consequently, enhancing mental health.

While this Research Topic does not present a comprehensive overview of the specifics of women's cycling, hopefully it will contribute to the advancement the field of “women's cycling” by prompting reflection among key stakeholders.
